# A Chaos Synchronization Diagnostic: Difference Time Series Peak Complexity (DTSPC)

**DOI:** 10.3390/e26121085

**Published:** 2024-12-12

**Authors:** Zhe Lin, Arjendu K. Pattanayak

**Affiliations:** 1 United World College Changshu China, Suzhou 215500, China; zlin22@uwcchina.org; 2 Department of Physics and Astronomy, Carleton College, Northfield, MN 55057, USA

**Keywords:** chaos, synchronization, entropy

## Abstract

Chaotic systems can exhibit completely different behaviors given only slightly different initial conditions, yet it is possible to synchronize them through appropriate coupling. A wide variety of behaviors—complete chaos, complete synchronization, phase synchronization, etc.—across a variety of systems have been identified but rely on systems’ phase space trajectories, which suppress important distinctions between very different behaviors and require access to the differential equations. In this paper, we introduce the Difference Time Series Peak Complexity (DTSPC) algorithm, a technique using entropy as a tool to quantitatively measure synchronization. Specifically, this uses peak pattern complexity created from sampled time series, focusing on the behavior of ringing patterns in the difference time series to distinguish a variety of synchronization behaviors based on the entropic complexity of the populations of various patterns. We present results from the paradigmatic case of coupled Lorenz systems, both identical and non-identical, and across a range of parameters and show that this technique captures the diversity of possible synchronization, including non-monotonicity as a function of parameter as well as complicated boundaries between different regimes. Thus, this peak pattern entropic analysis algorithm reveals and quantifies the complexity of chaos synchronization dynamics, and in particular captures transitional behaviors between different regimes.

## 1. Introduction

Certain dynamical systems have been known since Poincaré to be chaotic [1,2], i.e., they are highly sensitive to initial conditions and parameters. Classical models of deterministic chaos include the Lorenz system modeling weather [3] and the logistic map modeling population growth [4,5]. Dynamical systems can also display synchronization, when two or more different systems exhibit similar dynamics, either behaving identically as a limiting example, or more broadly possessing similarities in some key aspects, such as sharing the same frequencies or a constant phase lag [6]. Remarkably, though chaotic systems exhibit completely different behaviors with only slightly different initial conditions, adding a small amount of coupling can cause them to be synchronized. More broadly, chaos synchronization occurs when two (or many) chaotic systems (either identical, with the same equations, parameters and initial conditions, or dissimilar, with different equations, parameters or initial conditions) adjust a given property of their motion to a common behavior due to a coupling or a forcing (periodical or noisy) [7]. Chaos synchronization has found applications in a wide variety of fields such as electrical circuits [8,9,10,11], power systems [12,13,14], medicine [15,16], and chemical reactors [17,18].

Historically, chaos synchronization has been classified into different categories including complete synchronization, practical synchronization, and phase synchronization [19,20]. Such categorization, however, only provides qualitative classification of chaos synchronization, so research in quantitative complexity measurement of chaos synchronization started to develop over the last two decades. Classic methods of complexity measurement include phase portraits, bifurcation diagrams [21], and Lyapunov exponents [22], which require access to the dynamical equations. A growing body of work uses broader and more abstract techniques relying on entropic analysis such as using Kolmogorov entropy to quantify the level of unpredictability in a chaotic system over time [23], spectral entropy to diagnose multi-stable fractional-order chaotic systems [24], entanglement and relative entropy to investigate the chaotic behaviors in quantum systems [25], topological entropy to characterize synchronization in piecewise linear maps [26,27], and wavelet entropy to study brain electrical signals [28]. One advantage of these entropy-based analyses is that they can often be applied to systems where only the dynamical data are available, in the absence of the actual equations, such as in experimental situations, and the results are typically robust to the presence of noise in the data or the dynamics.

In this paper, we present numerical results from applying an entropic analysis to discrete peak patterns visible in the difference time series between systems to quantitatively measure chaos synchronization broadly, where such peak patterns should be visible across all basic models of chaos, including the Lorenz system, the Duffing oscillator, the Chua circuit, and the Rössler attractor [29]. Specifically, we use the paradigmatic model of the basic coupled Lorenz systems, applying entropic analysis to patterns extracted from peak dynamics. We show that this entropy-based analysis in quantitatively measuring chaos synchronization not only captures overall similarities between two synchronized systems but also reveals a diversity of possible synchronization, non-monotonic changes in behavior with respect to linear change in parameters during transition from one regime to another, and complicated boundaries between different synchronization regimes.

Below, in Section 2, coupled identical Lorenz systems with dissimilar initial conditions are introduced, as well as coupled dissimilar Lorenz systems with identical initial conditions, the synchronization effects of which vary with the change in coupling parameters. Section 3 describes the specific methodology of the new synchronization diagnostic, focusing on extracting from the raw time series information about patterns in the peak dynamics before computing the Shannon entropy of the pattern populations thus obtained. Results and findings about the coupled Lorenz systems after applying the new diagnostic are presented in Section 4 and are compared with the conventional methods, showing values of our new synchronization diagnostic. Finally, discussions and conclusions are presented in Section 5, revealing findings on other interesting behaviors and pointing out directions of further research.

## 2. Comparing Synchronization in Coupled Identical and Dissimilar Lorenz Systems

In this section, we discuss various details about different kinds of synchronization behaviors with the examples of coupled identical and dissimilar Lorenz systems, initially studied in weather forecasting. Under certain initial conditions and parameter values, the Lorenz system exhibits chaotic behavior. Before we introduce the more complex dynamics in the coupled dissimilar Lorenz systems, we first examine the coupled identical Lorenz systems.

### 2.1. Identical Lorenz Systems with Different Initial Conditions

Consider two Lorenz systems defined by

(1) $$\begin{array}{cc} \dot{x_{1}} & =\sigma_{1}\left(x_{2}-x_{1}\right)\\ \dot{x_{2}} & =-x_{1}x_{3}+\rho_{1}x_{1}-x_{2}\\ \dot{x_{3}} & =x_{1}x_{2}-\beta_{1}x_{3} \end{array}$$

and

(2) $$\begin{array}{cc} \dot{y_{1}} & =\sigma_{2}\left(y_{2}-y_{1}\right)\\ \dot{y_{2}} & =-y_{1}y_{3}+\rho_{2}y_{1}-y_{2}\\ \dot{y_{3}} & =y_{1}y_{2}-\beta_{2}y_{3} \end{array}$$

where $\sigma_{1}=\sigma_{2}=10,\rho_{1}=\rho_{2}=50,\beta_{1}=\beta_{2}=8/3$. The initial conditions for the first Lorenz are $x_{1}=0,x_{2}=1,x_{3}=0$; the initial conditions for the second Lorenz are $y_{1}=1,y_{2}=1,y_{3}=1$.

**Figure 1:**
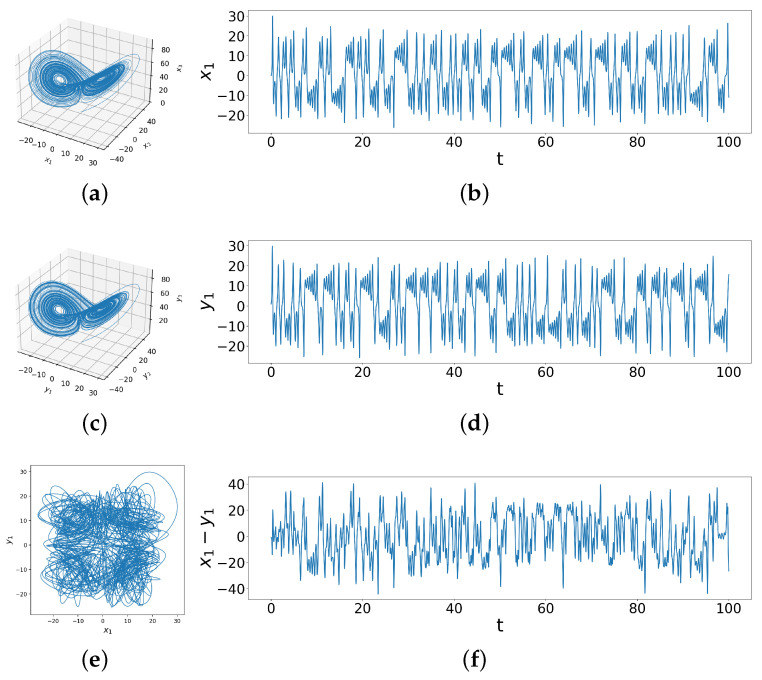
These figures show the behavior of two uncoupled identical Lorenz systems with different initial conditions. In (**a**,**b**) we see the phase space trajectory and time series for initial conditions $x_{1}=0,x_{2}=1,x_{3}=0$; (**c**,**d**) show the same for initial conditions $y_{1}=1,y_{2}=1,y_{3}=1$, while (**e**) shows $x_{1}$ vs $y_{1}$ phase space behaviors and (**f**) is the behavior of the difference time series $x_{1}-y_{1}$ for these two systems.

Throughout this paper, we use the ODEINT solver from SciPy, with time interval [0,100] and time step 0.001, in Python on a personal computing device to simulate solutions to the differential equations. Figure 1a,c show the 3D phase space diagrams of the two identical systems with different initial conditions. Although the two Lorenz systems have the same attractor, Figure 1e showing $x_{1}$ against $y_{1}$ makes it clear that the specific trajectories are quite different, demonstrated in time series data as well in Figure 1b,d and the difference time series of $x_{1}-y_{1}$ (Figure 1f), which behaves rather randomly. This sets our baseline to observe how coupling alters these differences.

We use the linear coupling model [7]

(3) $$\begin{array}{cc} \dot{x_{1}} & =\sigma_{1}\left(x_{2}-x_{1}\right)+d_{1}\left(y_{1}-y_{2}\right)\\ \dot{x_{2}} & =-x_{1}x_{3}+\rho_{1}x_{1}-x_{2}+d_{2}\left(y_{2}-x_{2}\right)\\ \dot{x_{3}} & =x_{1}x_{2}-\beta_{1}x_{3}+d_{3}\left(y_{3}-x_{3}\right)\\ \dot{y_{1}} & =\sigma_{2}\left(y_{2}-y_{1}\right)+d_{1}\left(x_{1}-y_{1}\right)\\ \dot{y_{2}} & =-y_{1}y_{3}+\rho_{2}y_{1}-y_{2}+d_{2}\left(x_{2}-y_{2}\right)\\ \dot{y_{3}} & =y_{1}y_{2}-\beta_{2}y_{3}+d_{3}\left(x_{3}-y_{3}\right) \end{array}$$

where $d_{1},d_{2},d_{3}$ are coupling strengths, which indeed yields that the trajectories of the two chaotic Lorenz systems do overlap. The parameters and initial conditions are the same as above: $\sigma_{1}=\sigma_{2}=10$, $\rho_{1}=\rho_{2}=50$, $\beta_{1}=\beta_{2}=8/3$, $x_{1}=0,x_{2}=1,x_{3}=0$ and $y_{1}=1,y_{2}=1,y_{3}=1$.

**Figure 2:**
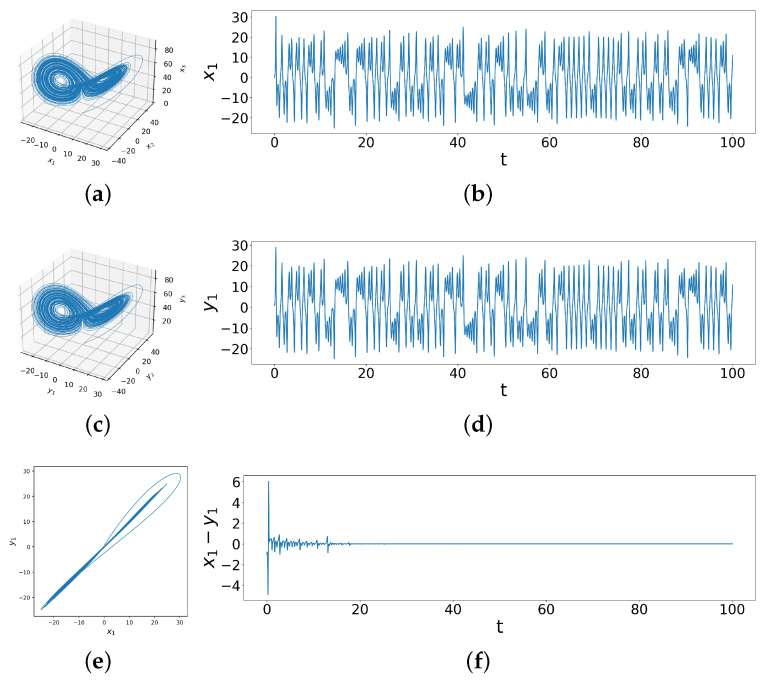
These figures show the behavior of two coupled identical Lorenz systems with different initial conditions under coupling strengths $d_{1}=3.0,d_{2}=1.2,d_{3}=0.0$. In (**a**,**b**) we see the phase space trajectory and time series for initial conditions $x_{1}=0,x_{2}=1,x_{3}=0$ under coupling; (**c**,**d**) show the same for initial conditions $y_{1}=1,y_{2}=1,y_{3}=1$ under coupling, while (**e**) shows $x_{1}$ vs $y_{1}$ phase space behaviors and (**f**) is the behavior of the difference time series $x_{1}-y_{1}$ for these two systems under coupling.

While Figure 2a,c look quite similar to the uncoupled systems (Figure 1a,c), the relationship between the two systems is now quite different, as visualized in the $x_{1}$ vs. $y_{1}$ phase space trajectories. In Figure 2e, the values of $x_{1}$ and $y_{1}$ are nearly identical, suggesting that the trajectories of the two systems almost completely overlap. Moreover, as shown in Figure 2f, the difference between $x_{1}$ and $y_{1}$ gradually reduce to zero over time. In fact, $x_{2}-y_{2}$ and $x_{3}-y_{3}$ are also decreasing down to zero. This proves that the trajectories of the two coupled Lorenz systems indeed gradually overlap, so complete synchronization between two identical chaotic systems with different initial conditions is achieved through linear coupling. Note that though the systems synchronize, each of them separately remain chaotic, as can be seen from the attractors in Figure 2a,c. The situation, however, becomes far more complicated in the case of coupled dissimilar Lorenz systems which possess identical initial conditions yet dissimilar parameters.

### 2.2. Dissimilar Lorenz Systems with Identical Initial Conditions

**Figure 3:**
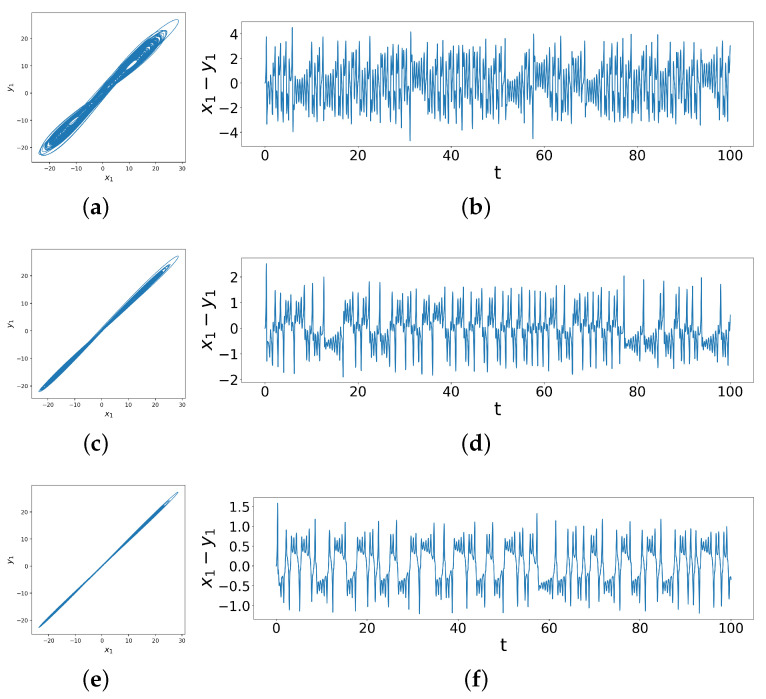
Phase space trajectories and $x_{1}-y_{1}$ difference time series of coupled dissimilar Lorenz systems with coupling strengths (**a**,**b**) $d_{1}=8.0,d_{2}=0.6$, (**c**,**d**) $d_{1}=8.0,d_{2}=4.0$, (**e**,**f**) $d_{1}=9.0$, $d_{2}=9.0$, showing weak synchronization under weak coupling and practical synchronization after strong coupling.

Above, we saw examples of non-synchronization before and complete synchronization after coupling. However, such clear distinctions do not completely cover the range of behaviors, as we show below; even a simple division into practical synchronization or phase synchronization does not suffice to describe the complex behavior seen between non-synchronization and complete synchronization. We can see this when we consider two slightly different Lorenz systems, one with parameters $\sigma_{1}=10,\rho_{1}=50,\beta_{1}=8/3$, the other with parameters $\sigma_{2}=10,\rho_{2}=40,\beta_{2}=8/3$. The initial conditions are now identical, i.e., $x_{1}=y_{1}=0,x_{2}=y_{2}=1,x_{3}=y_{3}=0$. Each of the two systems are chaotic, and they are unsynchronized. On introducing linear coupling, we see a wide range of different synchronization behaviors with more variety, as shown in Figure 3, compared to the coupled identical Lorenz systems.

In particular, we see in Figure 3 that as the coupling increases, the range of $x_{1}-y_{1}$ decreases from roughly ±4 to roughly ±2. Notice, in comparing Figure 3a–d with Figure 3e,f (at relatively different coupling strengths), we see that $x_{1}-y_{1}$ behaves similarly in phase space but displays more variety in the time series. Specifically, notice that there are peaks and dips above and below the horizontal line of $x_{1}-y_{1}=0$ in all three cases, but the last two cases also exhibit ringing patterns (corresponding to entrainment at certain differences) in practical synchronization, where the systems only display small variations in phase and amplitude, which means their trajectories’ difference converges to a neighborhood around the origin [20]. Moreover, notice that as the coupling strengths increase from the second case to the third case, these ringing patterns change character—specifically, oscillating for longer durations around locations further from the horizontal axis (zero difference). This change in ringing patterns as a function of coupling strengths is what distinguishes behaviors where phase-portraits could be a misleading indicator. We now quantitatively measure the behavior of the difference time series to more rigorously identify different synchronization patterns across changing coupling strengths, using an analysis of peak patterns via its computed Shannon entropy, a technique which we term the Difference Time Series Peak Complexity (DTSPC). This quantification allow us to map carefully, via a heat map of the DTSPC vs. coupling strengths, different kinds of synchronization and transitions as well as the complex shape of the transition regimes. We now turn to describing the method.

## 3. Methods: Quantitatively Measuring Synchronization

Although the time series patterns are quite different across the various types of synchronization shown in Figure 3, they all have the common element that the difference between $x_{1}$ and $y_{1}$ is centered around 0, i.e., the time series is centered around the horizontal axis $x_{1}-y_{1}=0$. We focus on behaviors relative to this horizontal axis, specifically considering the behavior of the ringing patterns previously discussed in the context of the difference time series for $x_{1}-y_{1}$. We count the number of peaks between zero-crossings in the difference time series, noting either the number of maxima if $x_{1}-y_{1}>0$ or the number of minima if $x_{1}-y_{1}<0$. This compresses the time series data into a list of the observed populations of number of consecutive peaks between zero-crossings in the original difference time series.

**Figure 4:**
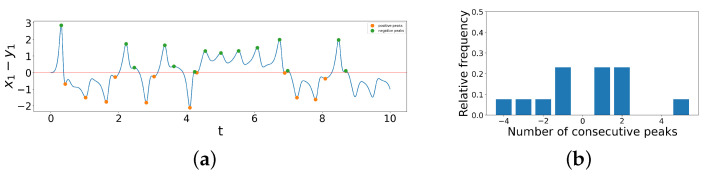
(**a**) Illustration of constructing peak series from difference time series for time interval [0,10] of a coupled dissimilar Lorenz system when $d_{1}=1.2,d_{2}=9.4$. (**b**) Bar chart of distribution of number of consecutive peaks in the difference time series.

We now examine the $x_{1}-y_{1}$ difference time series of time interval [0,10] of a coupled dissimilar Lorenz system when $d_{1}=1.2,d_{2}=9.4$, shown in Figure 4a, and apply our method to create a peak series out of it. We first note that $x_{1}-y_{1}$ starts at 0 at t=0, and the first zero-crossing occurs after one maximum, marked in green, above the central red line. Hence, our first element of the peak series is 1. Then, between the second and third zero-crossing, there are three minima, marked in orange, below $x_{1}-y_{1}=0$, so our second element of the peak series is −4. Similarly, we can convert the number of consecutive peaks between each zero-crossing into a numerical element in the peak series. The peak series, then, of this difference time series from time interval [0,10] is

1,−4,2,−2,2,−1,1,−1,5,−1,1,−3,2.

Note that the last two minima are not counted because there does not exist another zero-crossing until beyond t=10, which is not included in this truncated difference time series. To visualize the distribution of number of consecutive peaks in the list, we create a bar chart, as shown in Figure 4b below, where the *x*-axis denotes the elements in the peak series and the *y*-axis denotes the relative frequency.

**Figure 5:**
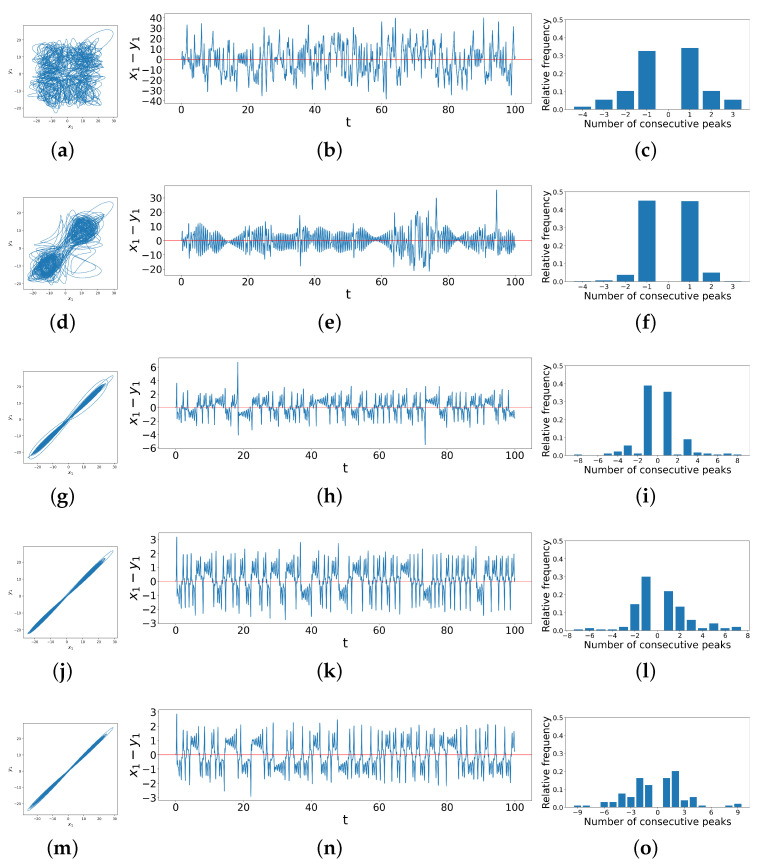
Phase space trajectories, $x_{1}-y_{1}$ difference time series and peak distribution bar charts for coupled dissimilar Lorenz systems with coupling strengths $d_{1}=1.2$ and (**a**–**c**) $d_{2}=0$, (**d**–**f**) $d_{2}=1.0$, (**g**–**i**) $d_{2}=6.4$, (**j**–**l**) $d_{2}=8.0$, and (**m**–**o**) $d_{2}=9.4$, showing correlations between phase space trajectories, difference time series and distribution of consecutive peaks.

Now, we extend the method illustrated using the truncated difference time series above to a longer time interval and a wider variety of cases, i.e., different coupling strengths $d_{1}$ and $d_{2}$, as shown in Figure 5 below. The figure shows the phase space trajectories (left column), the difference time series (middle column), and the distribution of consecutive peaks (right column) of coupled dissimilar Lorenz systems under $d_{1}=1.2$ and $d_{2}=0,1.0,6.4,8.0,9.4$, respectively.

As we look down the rows, the coupling strength $d_{2}$ increases while $d_{1}$ stays unchanged, and the phase portraits become more linear. Correspondingly, the bar charts in the right column show that the distribution of consecutive peaks in the difference time series also changes with the increase in $d_{2}$. When the system is not synchronized, there are no ringing patterns but only single spikes, so there are usually very few instances of more than one consecutive peak, as shown in Figure 5c. As the transition begins, the range of possible values for numbers of consecutive peaks increases, whence the bar charts show a corresponding wide range of values on the *x*-axis. At the early stage of such a transition, single peaks are still the majority, as shown in Figure 5f. We find here that the ringing pattern that exists often crosses the horizontal axis such that only the last peak in a ringing pattern counts as a single `peak’ by our algorithm. Then, as the system becomes more synchronized with the increase in coupling strengths, the ringing patterns diverge from the horizontal axis, so the consecutive peaks of all lengths are distributed more evenly, as shown in Figure 5o. All of these observations motivate us to quantify the complexity by computing the Shannon entropy of the distribution of peak populations [30], thus yielding the Difference Time Series Peak Complexity (DTSPC) metric,

(4) $$\zeta \left(d_{1},d_{2}\right)=-\sum_{i=1}^{n}p_{i}\log_{2}p_{i}$$

to quantitatively measure the level of synchronization.

Although normalized entropy is often used for statistical measures, we prefer unnormalized DTSPC to normalized DTSPC. The nature of our DTSPC technique, i.e., the fact that we utilized the natural peak pattern of difference time series to generate our peak series, results in varying possibilities of consecutive peaks under each pair of coupling strengths. Hence, normalization greatly affects the trend of color change in the heat map, as shown in Appendix A. After examining the phase space trajectories, difference time series and peak distribution bar charts corresponding to different regions in the heat maps, which we will further discuss in the next section, we believe that unnormalized DTSPC more accurately captures the trend of change in level of synchronization with the increase in coupling strengths.

## 4. Results

**Figure 6:**
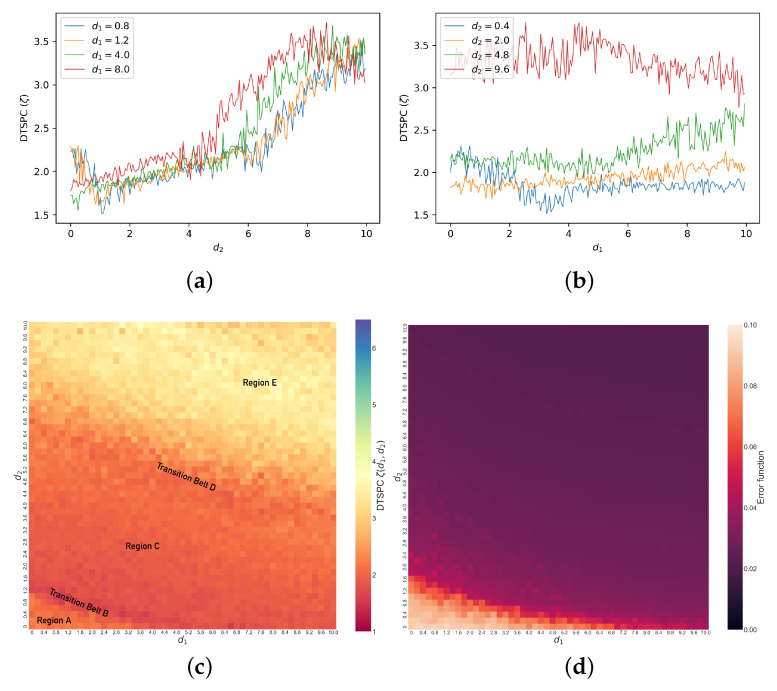
Line graphs of (**a**) ζ versus $d_{2}$ when $d_{1}=0.8,1.2,4.0,8.0$ and (**b**) ζ versus $d_{1}$ when $d_{2}=0.4,2.0,4.8,9.6$. (**c**) Heat map of $\zeta (d_{1},d_{2})$ (DTSPC) with changing coupling strengths in coupled dissimilar Lorenz systems. Region *A*: unsynchronized chaos. Transition Belt *B* and Region *C*: abrupt and then gradual decrease in $x_{1}-y_{1}$, enhanced synchronization. Transition Belt *D* and Region *E*: emergence and enhancement of separated ringing patterns, practical synchronization. (**d**) Heat map of error $e(d_{1},d_{2})$ with changing coupling strengths in coupled dissimilar Lorenz systems.

We now show how DSTPC ($\zeta (d_{1},d_{2})$) captures behavioral changes as $d_{2}$ increases for different constant $d_{1}$ values under the initial conditions $x_{1}=y_{1}=0,x_{2}=y_{2}=1,x_{3}=y_{3}=0$. The line graph of ζ versus $d_{2}$ when $d_{1}=1.2$ is plotted in Figure 6a. As we see in the graph, there is a significant drop in ζ between $d_{2}=0.0$ and $d_{2}=1.0$, a sudden increase between $d_{1}=1.0$ and $d_{1}=2.0$, a steady increase between $d_{2}=2.0$ and $d_{2}=6.0$, a sudden increase between $d_{2}=6.0$ and $d_{2}=7.0$, and a steady increase after $d_{2}=7.0$. Other colored lines in the figure also show ζ as a function of $d_{2}$ under other conditions—$d_{1}=0.8,4.0,8.0$, respectively. These four line graphs share similarities. The two respective periods of sudden and steady increases are present in all graphs. Moreover, when $d_{1}$ is larger, the initial sudden drop in ζ at $d_{2}\in \left[0,1\right]$ disappears, and there starts to emerge a final drop in ζ at large $d_{2}$ values. The gradual changes in the plots with the increase in $d_{1}$, as well as the changes in ζ with the increase in $d_{2}$ (Figure 6b), motivate us to create a heat map of $\zeta (d_{1},d_{2})$, i.e., displaying the DTSPC as a function of both coupling strength parameters.

In Figure 6c, we show the DTSPC heat map for the coupled dissimilar Lorenz systems under initial conditions $x_{1}=y_{1}=0,x_{2}=y_{2}=1,x_{3}=y_{3}=0$. The generation of such a heat map is within a reasonable time frame, i.e., between $10^{3}$ and $10^{4}$ seconds on a personal computing device using Python. We note that we have not conducted exhaustive convergence analysis for our results, since our goal is to explore if our technique can distinguish between different kinds of observed synchronization from time series rather than comprehensively studying the Lorenz system itself. To show the advantages and accuracy of our technique, we compare our DTSPC heat map against the conventional error function (the time-averaged distance between the oscillator state vectors defined by $e=\sqrt{{\left(x_{1}-y_{1}\right)}^{2}+{\left(x_{2}-y_{2}\right)}^{2}+{\left(x_{3}-y_{3}\right)}^{2}}$ [19]) heat map shown in Figure 6d. We see that our DTSPC heat map captures not only the main transitions in the bottom-left corner of the error function heat map, but it also reveals details in different levels of synchronization across the rest of the heat map. We find distinct regimes of different levels and types of synchronization via our DTSPC heat map. The color coding for ζ ranges from warmer dark red to light yellow, the warmer color representing a smaller ζ and hence a less complex set of data from the synchronization dynamics. Though the range of ζ is roughly between 1 and 4, we choose the range [1,6] for the color bar for convenience in comparison with the coupled identical case presented later. The heat map starts from orange in the bottom-left corner, indicated as Region *A*, and transitions through the dark red Belt *B*, beyond which is Region *C*, where $\zeta (d_{1},d_{2})$ changes gradually. Beyond this Region *C* is the orange-yellow Transition Belt *D*, separating it from Region *E* above, which is bright yellow.

**Figure 7:**
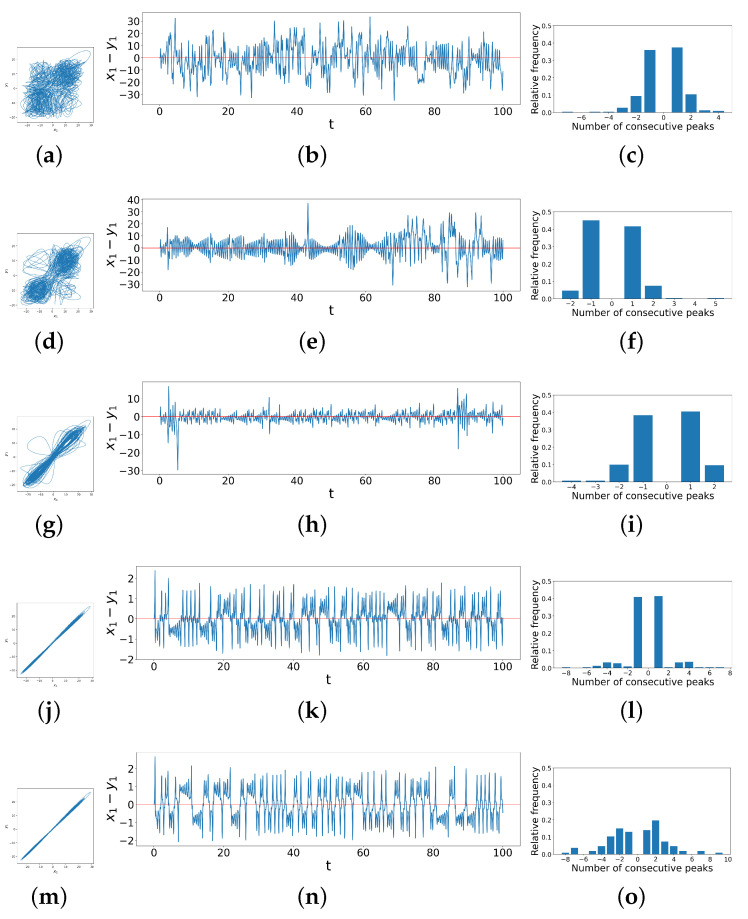
Phase space trajectories, $x_{1}-y_{1}$ difference time series and peak distribution bar charts for different regimes of coupled dissimilar Lorenz systems. (**a**–**c**) correspond to a case in Region *A* in Figure 6c when $d_{1}=1.0,d_{2}=0.4$; (**d**–**f**) correspond to Transition Belt *B* in Figure 6c when $d_{1}=0.6,d_{2}=1.2$; (**g**–**i**) correspond to Region *C* in Figure 6c when $d_{1}=1.2,d_{2}=2.6$; (**j**–**l**) correspond to Transition Belt *D* in Figure 6c when $d_{1}=8.8,d_{2}=4.0$; (**m**–**o**) correspond to Region *E* in Figure 6c when $d_{1}=2.2,d_{2}=9.0$.

While it is important to emphasize that we can construct such a heat map given just the peak data without access to either the differential equations or the time series themselves, to understand what these various regimes represent, in Figure 7, we explore details of these different synchronization behaviors, showing examples from each region of the heat map. Region *A* represents chaotic dynamics for any measure of difference between the two systems, and we can see this, for example, in Figure 7a, in which the phase space trajectories of $y_{1}$ vs. $x_{1}$ are chaotic and patternless. Figure 7b also displays the $x_{1}-y_{1}$ difference, which wanders between ±30. Because of this lack of pattern in the difference time series, it frequently crosses zero, so observations of the number of consecutive peaks between zero-crossings are therefore mainly centered at −1 and 1, as shown in Figure 7c, resulting in a low ζ. Transition Belt *B* signals a rapid onset of synchronization as a function of coupling strengths. In the phase-portrait (Figure 7d), we see that the in-phase component of the trajectories—that is, trajectory lines being traced from the bottom-left to top-right diagonal—increases, while the out-of-phase component—that is, trajectory lines being traced from the bottom-right to top-left—decreases. This also means that $x_{1}$ and $y_{1}$ are more directly correlated, and in general, the phase-space trajectories become more linear. We see this in the difference time series (Figure 7e), which abruptly decreases to lie between ±20 most of the time and is more symmetric around zero. This leads to the data in the bar chart (Figure 7f) becoming even more centered at −1 and 1 consecutive peaks, resulting in a decrease in ζ.

In Region *C*, the differences continue to decrease, with the in-phase component of the phase space trajectories (Figure 7g) continuing to become sharper and more linear. In the difference time series (Figure 7h), there start to appear some ringing patterns above and below the horizontal axis, though not yet very obvious. The difference between $x_{1}$ and $y_{1}$ further decreases to lie roughly between ±10. Transition Belt *D* is where practical synchronization becomes rather apparent. As shown clearly in the phase space trajectories (Figure 7j), $x_{1}$ and $y_{1}$ follow a linear relationship. Accordingly, the difference between these two observables (Figure 7k) reduces to between ±3, and clear ringing patterns start to appear that lie strictly above and below the horizontal axis of $x_{1}-y_{1}=0$. However, before this clear separation above and below the horizontal axis, we also see some small-scale dynamics in this difference variable, which presents as `signal fuzziness’ and a `stickiness’ when this difference crosses zero, resulting from the slight entanglement of the two oscillators’ trajectories when they are exchanging positions in phase space during synchronization, and often resulting in a small single peak for our criterion. This results in many −1 and 1 consecutive peaks (Figure 7l), so the ζ remains low. Finally, Region *E* is where the two systems are more synchronized, as the ringing patterns appear further apart from each other, shown in Figure 7n, with fewer crossings at zero. This indicates that there is less stagnation during a phase change, such that the number of −1 and 1 consecutive peaks reduces. Thus, the data are more spread out in the bar chart, as shown in Figure 7o, and ζ consequently experiences a significant increase. Note that there is a region of slightly darker yellow, or light orange, in the top-right corner of Region *E*. This slight drop in ζ, also reflected in Figure 6a when $d_{1}=4.0,8.0$, is a result of a reduced range in the numbers of consecutive peaks as the coupling strengths reach large values around 10, leading to smaller entropy values. Thus, to summarize, ζ starts off low in Region *A*, then decreases and increases with the increase in coupling strengths, as before for identical systems. However, the transition to synchronization now carries more regions of distinct dynamical regimes: Transition Belt *B*, Region *C*, and Transition Belt *D*. Within them, ζ first decreases a little, then increases, and slightly decreases again, each part corresponding to different dynamics of the system, as we have just seen. This shows again the value of using ζ to quantitatively identify and classify synchronization behaviors.

**Figure 8:**
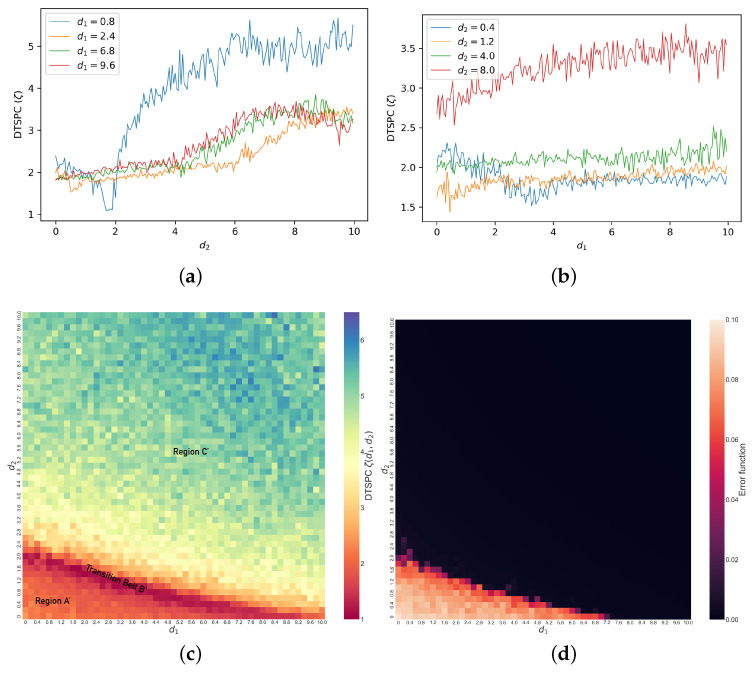
Line graphs of (**a**) ζ versus $d_{2}$ when $d_{1}=0.8,2.4,6.8,9.6$ and (**b**) ζ versus $d_{1}$ when $d_{2}=0.4,1.2,4.0,8.0$. (**c**) Heat map of ζ (DTSPC) with changing coupling strengths in coupled identical Lorenz systems, showing unsynchronized chaos in Region $A^{\prime }$, transition to synchronization in Transition Belt $B^{\prime }$, and complete synchronization in Region $C^{\prime }$. (**d**) Heat map of error function $e(d_{1},d_{2})$ with changing coupling strengths in coupled identical Lorenz systems.

In Figure 8c, we show the values of $\zeta (d_{1},d_{2})$ with changes in $d_{1}$ and $d_{2}$ in the coupled identical Lorenz systems with initial conditions $x_{1}=0,x_{2}=1,x_{3}=0,y_{1}=y_{2}=y_{3}=1$. Comparing against the error function heat map to its right, our DTSPC heat map reveals more clearly a gradual transition in ζ values across regimes of synchronization, indicating diverse synchronization behaviors with changes in coupling parameters. In particular, we can identify different regimes across the map based on the change in color patterns in the heat map. Specifically, moving diagonally starting from the bottom left corner, we label the dominantly orange-red region as Region $A^{\prime }$; this is followed by a distinct thin ridge of dark red, which we label as Transition Belt $B^{\prime }$. Beyond this Transition Belt $B^{\prime }$ is Region $C^{\prime }$, where the color steadily changes from yellow to bluish-green. We note immediately that we can visually identify a speckled pattern, suggesting that the transition need not be smooth as a function of coupling. We also see visually that these transitions are less sensitive to coupling (are slower) in Regions $A^{\prime }$ and $C^{\prime }$, while they are more rapid in Transition Belt $B^{\prime }$.

**Figure 9:**
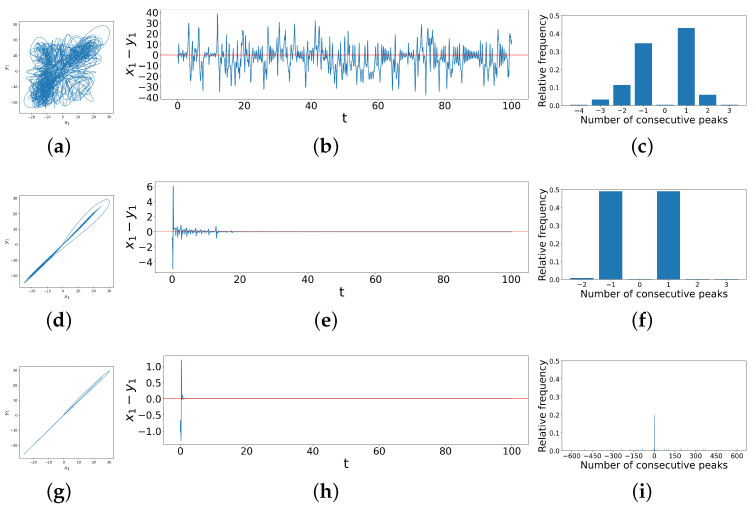
Phase space trajectories, $x_{1}-y_{1}$ difference time series and peak distribution bar charts for different regimes of coupled identical Lorenz systems. (**a**–**c**) correspond to a case in Region $A^{\prime }$ in Figure 8c when $d_{1}=1.4,d_{2}=0.8$; (**d**–**f**) correspond to Transition Belt $B^{\prime }$ in Figure 8c when $d_{1}=3.0,d_{2}=1.2$; (**g**–**i**) correspond to Region $C^{\prime }$ in Figure 8c when $d_{1}=5.0,d_{2}=5.0$.

As before, we specify the corresponding dynamical behaviors of the system under different regimes by examining examples from each, as shown in Figure 9. The first example is when $d_{1}=1.4,d_{2}=0.8$ in Region $A^{\prime }$, shown in Figure 9a–c. Region $A^{\prime }$ represents unsynchronized chaos, where $x_{1}$ and $y_{1}$ display the random relationship shown in the phase space trajectories (Figure 9a). Similarly, in the time series (Figure 9b), the difference between $x_{1}$ and $y_{1}$ remains significant and irregular. This results in its constant crossing of zeros, with few segments of the time series showing larger numbers of consecutive peaks. Thus, there are mainly 0 or 1 consecutive peaks according to our criterion, indeed, as shown in Figure 9c, which leads to the low ζ value. In Transition Belt $B^{\prime }$, dynamics change quickly with parameters, as shown in Figure 9d–f when $d_{1}=3.0,d_{2}=1.2$. The phase space trajectories (Figure 9d) show that the relationship between $x_{1}$ and $y_{1}$ becomes mainly linear, and in time series (Figure 9e), accordingly, the difference between $x_{1}$ and $y_{1}$ gradually decreases to zero, where there remains some bursts at the beginning in the first 20 time steps. This time series frequently crosses the horizontal axes such that the count for consecutive peaks remains even more concentrated at 0 or 1 than in Region $A^{\prime }$, so ζ for systems in the Transition Belt *B* is even lower. The third example when $d_{1}=5.0,d_{2}=5.0$ is chosen from Region $C^{\prime }$ and represents complete synchronization. In the phase space trajectories (Figure 9g), the relationship between $x_{1}$ and $y_{1}$ is even more strictly linear, and the difference time series (Figure 9h) reduces to zero immediately after the two Lorenz systems are coupled. However, there could be nuanced and extremely frequent ringing patterns above or below the zero line (red horizontal line), as the number of consecutive peaks can range from −600 to 600, as shown in Figure 9i. Hence, this analysis reveals overall that as a function of coupling parameters, the dynamical synchronization as quantified by ζ starts low, and then with the increase in coupling strengths, it experiences first a decrease then an increase, which matches the general trend of the heat map (Figure 8c). In summary, we see that coupling in general increases synchronization, though the transition can be abrupt and non-monotonic in this case, and that the DTSPC captures this appropriately.

It is worth noting that our results show that the relationship between the level of synchronization and the value of $\zeta (d_{1},d_{2})$ is rather unconventional, namely, when the entropy ζ increases, the level of synchronization increases as well. This is in fact due to the reason that practical synchronization (Figure 7n), the ultimate state for coupled dissimilar systems, has ringing patterns around 0, but complete synchronization (Figure 9h), the ultimate state for coupled identical systems, might not, resulting in a lack of zero-crossings and therefore huge values in peak series, leading to a larger ζ value. Hence, $\zeta (d_{1},d_{2})$ is not a direct quantification of synchronization that draws a linear relationship between the two, but is instead a measure of the complexity of the differences in trajectories between two synchronized chaotic systems.

**Figure 10:**
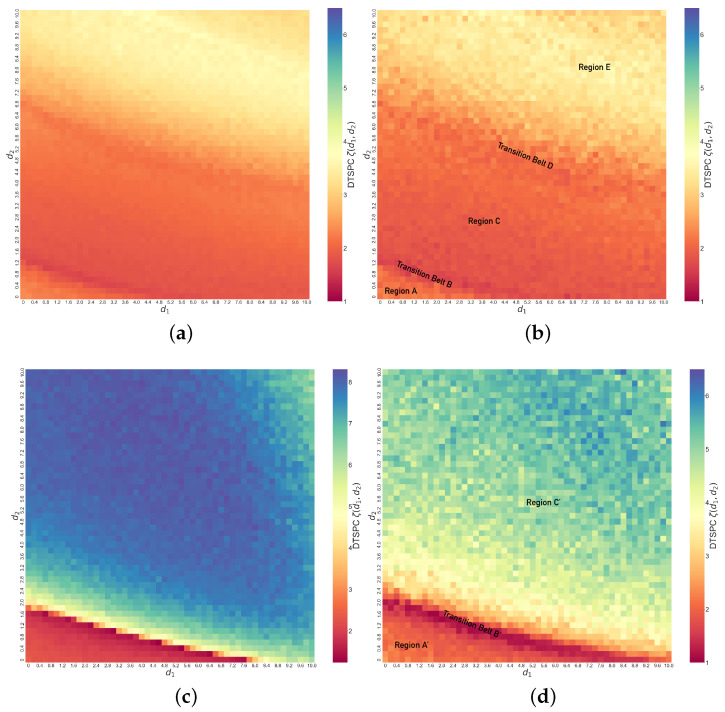
(**a**) Heat map for coupled dissimilar Lorenz systems with $\zeta (d_{1},d_{2})$ computed using time interval [0,1000] with original time step 0.001. (**b**) Original heat map for coupled dissimilar Lorenz systems (same as Figure 6c, with time interval [0,100] and time step 0.001). (**c**) Heat map for coupled identical Lorenz systems with $\zeta (d_{1},d_{2})$ computed using time interval [0,1000] with original time step 0.001. (**d**) Original heat map for coupled identical Lorenz systems (same as Figure 8c, with time interval [0,100] and time step 0.001).

We also note that both heat maps (as shown in Figure 6c and Figure 8c) show that the synchronization is not locally smooth as a function of coupling strength, given the speckled pattern or occasional grids visibly brighter or darker than their surroundings. However, if we increase the time interval for which we generate the peak series and to which we apply our DTSPC technique, the speckled patterns become significantly less obvious. Below, we show the DTSPC heat maps with a time interval 10 times longer (t∈[0,1000]) than what we took before (t∈[0,100]). Notice that we use the same color bar scale for the two heat maps for coupled dissimilar Lorenz (Figure 10a,b). Figure 10a is in fact an enhanced version of Figure 10b with more refined transition boundaries and less obvious speckled patterns. The case is similar for coupled identical Lorenz (Figure 10c,d). You may notice that in Figure 10c, we allow for a bigger scale of ζ values on the color bar because some values reach beyond 6.5, which was our previous color bar maximum. This, we believe, is due to the fact that complete synchronization is achieved after coupling identical Lorenz systems, so a longer time interval taken will result in consecutive peaks even more dispersed than the case in Figure 9i. However, a darker color in the region for complete synchronization does not affect how accurate our DTSPC heat map, using any sufficiently long time interval, can capture the overall regimes of different synchronization behaviors. Heat maps generated after taking a 10 times longer time interval for analysis prove to be more precise, yet we would like to point out that the computational demand is 10 times higher than our previous heat maps generated using a shorter time interval, while both are accurate enough for reflecting and distinguishing the diverse dynamics at different levels of synchronization.

With our DTSPC technique, we can also investigate the synchronization of coupled Lorenz systems with other different initial conditions or different parameters. The DTSPC metric, while detecting slight fluctuations in the synchronization dynamics for systems with other initial conditions, reveals that initial conditions do not affect synchronization regimes much, while the systems’ parameter values do have a nontrivial impact. Further detailed discussion of these can be found in Appendix B. However, we would like to emphasize that these discoveries are beyond the scope of this paper, which aims to provide a new metric to quantitatively measure chaos synchronization and verify its accuracy.

## 5. Discussion and Conclusions

In this paper, we computed the Shannon entropy of peak population distributions in the difference time series of chaotic coupled Lorenz systems, introducing a Difference Time Series Peak Complexity (DTSPC) diagnostic for chaos synchronization. This digitizes a characteristic ringing pattern that changes as a function of parameter in the dynamics of the difference variable. We find that the DTSPC entropic analysis of extracted peak data compresses useful information and in particular is able to distinguish and quantitatively measure via a single metric the differences in system behavior during the transition from non-synchronization to synchronization. We are thus able to more systematically study variations in synchronization across parameter values and initial conditions, and present both the range of behaviors and its parametric dependence via a single heat map. In particular, in the test cases of our coupled identical and dissimilar Lorenz systems, we can distinguish a variety of synchronization behaviors, clarifying that though system behaviors can be macroscopically classified into non-synchronization or complete synchronization, such rough classification omits detailed behaviors, including non-monotonic parameter dependence. We again emphasize that these behaviors are not visible when examining phase space trajectories and can be constructed using substantially less access to the complete time series; these results are also robust to minor noisy perturbations.

In conclusion, the success of our Difference Time Series Peak Complexity (DTSPC) diagnostic in creating digitized representation of diverse synchronization behaviors from phase space trajectories and difference time series demonstrates the effectiveness of using Shannon entropy to quantitatively measure chaos in systems with peak dynamics, specifically utilizing the statistical information behind peak distribution. For further development of this research, one can test and evaluate the applicability of entropy and its variations on chaotic systems without significant peak dynamics or of higher dimensions. Additionally, the speckled patterns in the heat maps (Figure 6c and Figure 8c) suggest complex boundaries between different behaviors as a function of coupling parameters when using a finite time interval; however, from Figure 10a,c, we conclude that such complex boundaries become smoother when using a longer time interval, though doing so is computationally expensive. Finally, while we note that it would be possible to fit the boundaries between regimes with approximate curves that would allow us to extract parametric boundaries, it is not our goal to specify these details for any specific system. We hope that this inspires those studying or using synchronization in Lorenz—and other—systems to further develop the connection of our technique to system particulars and analytical details.

## Figures and Tables

**Figure A1:**
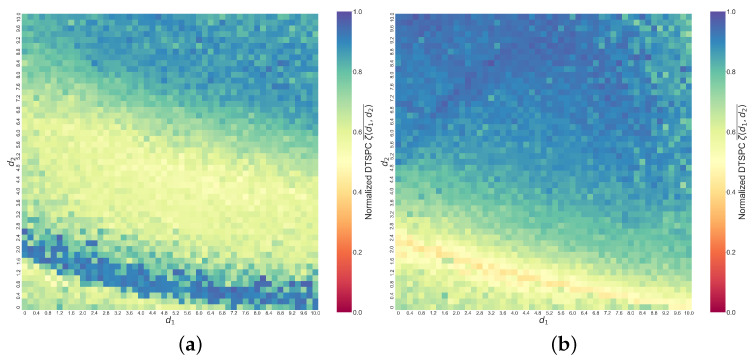
Heat maps of normalized DTSPC ($\bar{\zeta (d_{1},d_{2})}$ for (**a**) coupled dissimilar Lorenz systems with initial conditions $x_{1}=y_{1}=0,x_{2}=y_{2}=1,x_{3}=y_{3}=0$ and (**b**) coupled identical Lorenz systems with initial conditions $x_{1}=0,x_{2}=1,x_{3}=0,y_{1}=y_{2}=y_{3}=1$. Initial conditions are consistent with those used throughout the paper.

**Figure A2:**
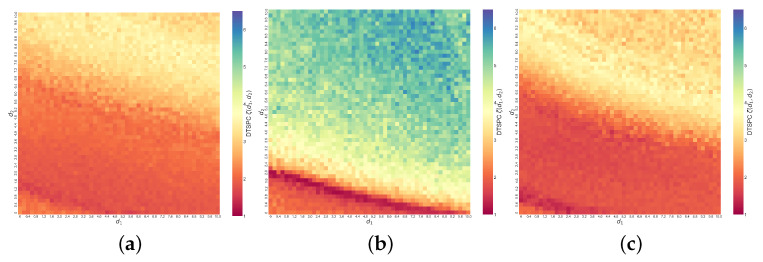
Heat maps of (**a**) coupled dissimilar Lorenz systems with initial conditions $x_{1}=0,x_{2}=1$, $x_{3}=0,y_{1}=5,y_{2}=6,y_{3}=5$, (**b**) coupled identical Lorenz systems with initial conditions $x_{1}=3$, $x_{2}=1,x_{3}=2,y_{1}=8,y_{2}=6,y_{3}=5$ and (**c**) coupled dissimilar Lorenz systems with parameters $\rho_{1}=28,\rho_{2}=29$ and same initial conditions $x_{1}=y_{1}=0,x_{2}=y_{2}=1,x_{3}=y_{3}=0$.

## Data Availability

The computer code and data used in this manuscript are available from the authors on reasonable request.

## Abbreviations

The following abbreviations are used in this manuscript:

DTSPC	Difference Time Series Peak Complexity

## Appendix A. Heat Maps of Normalized DTSPC

Based on the definition of normalized entropy, the normalized DTSPC is defined by

$$\begin{array}{cc} \bar{\zeta (d_{1},d_{2})} & =\frac{-\sum_{i=1}^{n}p_{i}\log_{2}p_{i}}{-\sum_{i=1}^{n}\frac{1}{n}\log_{2}\frac{1}{n}}\\ \; & =-\frac{\sum_{i=1}^{n}p_{i}\log_{2}p_{i}}{\log_{2}n}\\ \; & =\frac{\zeta (d_{1},d_{2})}{\log_{2}n} \end{array}$$

Below, we show the heat map figures we obtain by plotting $\bar{\zeta (d_{1},d_{2})}$ over $d_{1}$ and $d_{2}$. Comparing Figure 1a with Figure 6c and Figure 1b with Figure 8c, we notice that the different regimes in the heat maps of unnormalized DTSPC are equally distinguished in the normalized DTSPC, but the coloring in the normalized DTSPC heat maps is inconsistent with the dynamics of the Lorenz systems. For example, both the thin transition belt at the bottom of Figure 1a and the top-right region are colored in blue, meaning that they have similar $\bar{\zeta (d_{1},d_{2})}$ values, yet in Section 4, we could see that the two regions represent very different behaviors of the coupled dissimilar Lorenz systems, corresponding to Figure 7d–f and Figure 7m–o, respectively. Similar issues arise when we notice that both regions below and above the light orange transition belt in Figure 1b are colored in the same green, yet they also represent different behaviors of the systems. The colors in the heat maps of normalized DTSPC do not change uni-directionally with the change in level of synchronization, leading to ambiguity in interpreting the behaviors of the systems using the heat maps. Hence, we prefer unnormalized DTSPC.

## Appendix B. Heat Maps Generated Under Other Initial Conditions and Parameters

Below, we present our findings after generating heat maps of $\zeta (d_{1},d_{2})$ for coupled dissimilar Lorenz systems with other initial conditions (Figure 2a), coupled identical Lorenz systems with other initial conditions (Figure 2b) and coupled dissimilar Lorenz systems with different parameter values than what we have used throughout this paper (Figure 2c). Interestingly, Figure 2a,b are generally similar to the heat maps generated using our original initial conditions, where although locally there are different speckled patterns, the overall trends of coloring of the heat maps are preserved. This reveals that the differences in initial conditions barely affect the dynamics of synchronization of coupled Lorenz systems as long as the coupling method and strengths are kept identical. Figure 2c is the heat map for coupled dissimilar systems with parameters $\rho_{1}=28,\rho_{2}=29$, while we have used $\rho_{1}=50,\rho_{2}=40$ for coupled dissimilar Lorenz systems throughout the paper. This heat map presents a slightly different overall trend in the coloring than that in Figure 6c, the former with a larger orange region in the top-right corner. After verifying using the phase space trajectories, the difference time series and the peak distribution bar charts, we confirm that the orange region in the top-right corner in Figure 2c does correspond to when there start to occur more single peaks with large coupling strengths.
